# Pattern of and clinicopathologic risk factors for lateral lymph node metastases in papillary thyroid carcinoma patients with lateral cervical lymphadenopathy

**DOI:** 10.1097/MD.0000000000012263

**Published:** 2018-09-07

**Authors:** Yanping Gong, Jing Yang, Shuping Yan, Anping Su, Feng liu, Rixiang Gong, Jingqiang Zhu, Zhihui Li

**Affiliations:** Thyroid and Parathyroid Surgery Center, West China Hospital, Sichuan University, No. 37 Guo Xue Xiang, Chengdu, Sichuan, China.

**Keywords:** lateral neck dissection, lymph node metastases, papillary thyroid carcinoma, pattern, risk factor

## Abstract

The surgical extension of lateral neck dissection (LND) in papillary thyroid carcinoma (PTC) with clinical lateral lymph node metastases (LLNM) remains controversial. The aim of this study was to explore the pattern of and clinicopathologic risk factors for LLNM in PTC with clinical unilateral LND to determine the rational extent of therapeutic LND.

This retrospective study reviewed the records of 246 consecutive patients with PTC who simultaneously underwent total thyroidectomy, bilateral central lymph node dissection, and unilateral therapeutic LND. The frequency and pattern of LLNM were analyzed.

Grossly, LLNM were present in 80.9% of patients, and level II to V lymph node metastases (LNM) were present in 45.9%, 62.6%, 56.1%, and 11.8% patients, respectively. Superior tumor location, extrathyroidal extension, and ipsilateral, contralateral, and bilateral central LNM (CLNM) were independent risk factors for gross LLNM. Age ≥45 years, superior lobe tumors, extrathyroidal extension, and ipsilateral and contralateral CLNM were independent risk factors for level II LNM. Age ≥45 years, superior and middle lobe tumors, extrathyroidal extension, and ipsilateral CLNM were independent risk factors for level III LNM. Superior lobe tumors and ipsilateral, contralateral, and bilateral CLNM were independent risk factors for level IV LNM. Only contralateral CLNM was an independent risk factor for level V LNM.

In PTC patients with clinical LLNM, the predominant sites of LLNM were levels II to IV and not level V. Therapeutic elective LND should include the lateral nodal levels associated with independent risk factors, especially superior tumors location and CLNM.

## 1 Introduction

Thyroid cancer is the most common endocrinal tumor, and its prevalence is increasing worldwide.^[1]^ As definition, differentiated thyroid carcinoma (DTC) includes papillary thyroid carcinoma (PTC) and follicular thyroid carcinoma that origins from follicular cell. Among DTC, PTC is the first common thyroid malignancy. Although DTC has an excellent prognosis, cervical lymph node metastases (LNM) occur frequently, especially in patients with PTC.^[2,3]^ Previous studies have identified LNM as an independent risk factor for local recurrence,^[4–7]^ and emerging evidence from large population-based studies indicates a decrease in disease-free survival rate and an increase in mortality associated with regional LNM.^[7–10]^ Although the majority of LNM are within the central compartment of the neck, approximately 15% occur in the lateral neck and can be detected by ultrasonography (US).^[11]^

Preoperative imaging examinations are useful to identify lateral LNM (LLNM), especially US and computed tomography (CT). The sensitivity and specificity of preoperative US to detect LLNM are as high as 70% and 82%, respectively, and for preoperative CT are as high as 97% and 90%, respectively.^[12]^ There is a consensus that therapeutic lateral neck dissection (LND) should be performed in patients with PTC and clinical LLNM based on palpation or imaging examination.^[13,14]^

Despite the agreement, the surgical scope of LND remains controversial. Conservative LND may increase the lymph node recurrence rate and disease-specific mortality because some occult lymph node metastases cannot be detected preoperatively. Conversely, radical LND can lead to clinically important postoperative complications such as injury to the spinal accessory nerve or the cervical plexus.^[15–18]^ Therefore, appropriate and effective therapeutic LND is crucial.

Most previous studies have explored the risk factors for LLNM at gross levels or at level II or/and level V. To our knowledge, few studies have explored the risk factors for LLNM at each nodal level. Furthermore, few studies have investigated the risk value of central LNM (CLNM) with separation into ipsilateral, contralateral, and bilateral compartments for LLNM. This study aimed to explore the frequency and pattern of LLNM, and the clinicopathologic risk factors (especially location and CLNM) for LLNM of each level (II–V) in PTC patients with clinical unilateral LNM. The findings could indicate the rational extent of therapeutic LND in the management of PTC, especially selective therapeutic LND.

## 2 Patients and methods

### 2.1 Study population

This retrospective study reviewed the medical records of consecutive patients with histologically proven PTC who underwent simultaneous total thyroidectomy (TT), bilateral central neck dissection (CND), and unilateral (ipsilateral to the largest primary tumor) LND (ranging from level II to V). The study was conducted in the Department of Thyroid and Breast Surgery, West China Hospital of Sichuan University, between January, 2014 and August, 2017. In all, 246 patients were enrolled in the study. All patients underwent preoperative physical examination, high-quality thyroid US, CT, and US-guided fine needle aspiration biology (FNAB) of the largest primary tumor. No patients presented with preoperative hoarseness indicating recurrent laryngeal nerve paralysis.

Patients were included in the study if they had clinical ipsilateral LLNM based on preoperative palpation, US, CT, or FNAB. The final diagnosis of primary tumors and cervical LNM was based on pathological examination of surgical specimens. Patients were excluded if they had thyroid carcinoma with mixed histology, re-operation, or undivided lymph node specimens. The institutional review board of the West China Hospital of Sichuan University approved the study. Written informed consent was obtained from all patients before enrolment in the study.

### 2.2 Tumor and lymph node classification

The diameter and location of the largest primary tumor within the thyroid was determined from pathology reports or imaging results, most commonly US. The location of the tumor was classified based on which third of the affected thyroid lobe was involved (superior, middle, or inferior). Lesions confined to the isthmus were treated as middle third lesions. If a tumor extended into an adjacent lobe, it was categorized based on all thirds involved.^[19]^

Removal of the entire thyroid gland was performed first, followed by bilateral CND and unilateral LND. The maximum extent of CND was the hyoid bone superiorly, the innominate vein inferiorly, and the carotid sheaths laterally. Central lymph node (CLN) specimens were first classified as ipsilateral paratracheal, pretracheal, or contralateral paratracheal. Next, the prelaryngeal, pretracheal, and ipsilateral paratracheal lymph nodes were defined as ipsilateral CLN and the contralateral paratracheal lymph node as contralateral CLN, in accordance with the definition of laterality proposed by Keum et al.^[20]^ The surgeon separately removed each of these categories of nodes. The LND was performed in the usual fashion from level II to level V, sparing the internal jugular vein, spinal accessory nerve, and sternocleidomastoid muscle. The surgeon also separated the LND specimens according to neck levels. All thyroid and LND specimens were sent to the pathology department for fixation in paraffin and histological analysis.

Information on the following clinicopathologic factors was obtained: sex, age, size of the primary tumor(s), location of the largest primary tumor, multifocality, presence of coexistent lymphocytic thyroiditis, extrathyroidal extension, and the extent of lymph node metastasis.

### 2.3 Statistical analysis

Categorical variables were analyzed using Pearson chi-square test, and continuous variables were analyzed using the Student *t* test or the Wilcoxon rank-sum test. Binary logistic regression analysis was used for the multivariate analysis of categorical variables. All *P* values were 2-sided, and a *P* value of <.05 was considered statistically significant. Statistical analysis was performed using STATA 12.0 software (Stata Corporation, College Station, TX).

## 3 Results

### 3.1 Patient and tumor characteristics

In all, 246 patients were enrolled in the study. Out of these, 55 (22.4%) were male and 191 (77.6%) were female. The median age was 42.6 years (range 14–80 years). The mean size of the largest primary thyroid tumor was 21.3 mm (range 4–75 mm). A summary of patient and tumor characteristics is shown in Table 1. The patients with gross LLNM and level II, level III, and level IV LNM, but not level V LNM, were younger compared with patients without LNM (Tables 2–6). The mean sizes of the largest primary tumor of patients with gross LLNM and each level of LNM were larger compared with those of patients without LNM (Tables 2–6).

**Table 1:**
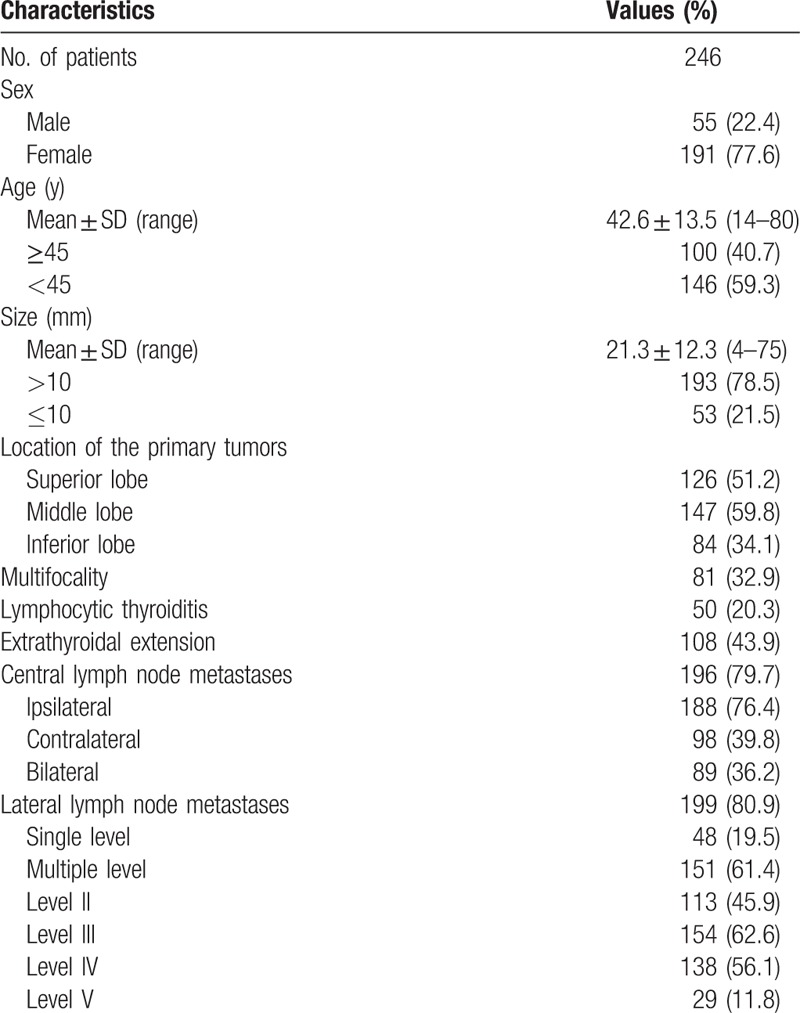
Demographics and clinical characteristics of patients.

**Table 2:**
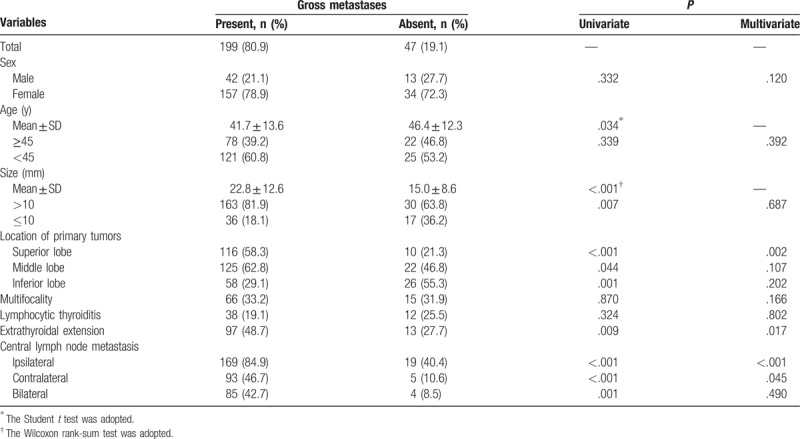
Analysis of risk factors related to gross lateral lymph node metastases.

**Table 3:**
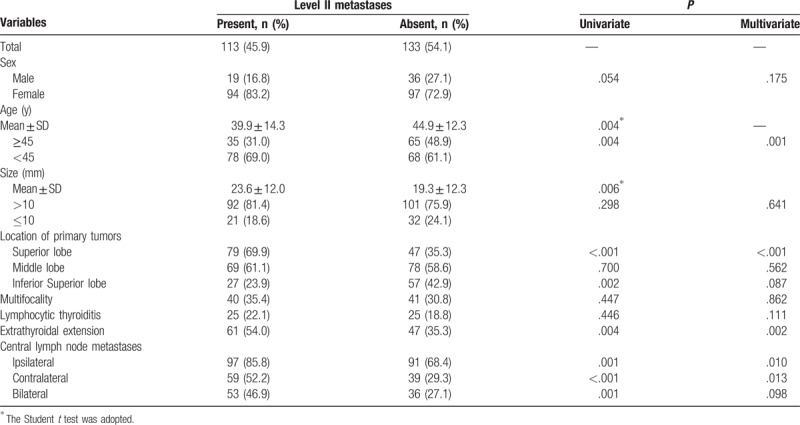
Analysis of risk factors related to level II lymph node metastases.

**Table 4:**
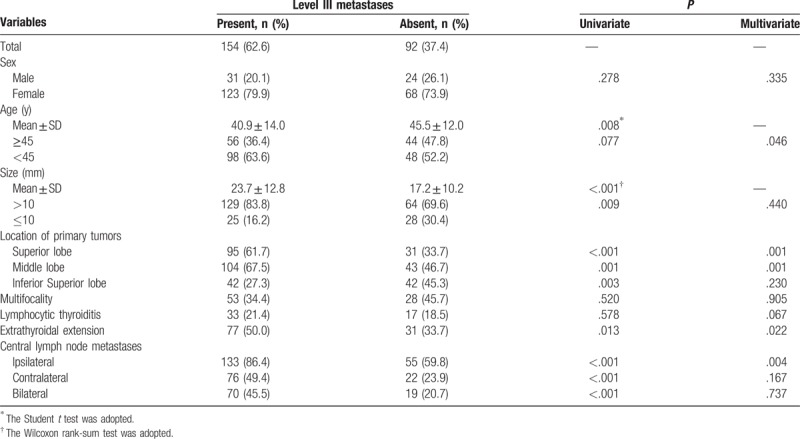
Analysis of risk factors related to level III lymph node metastases.

**Table 5:**
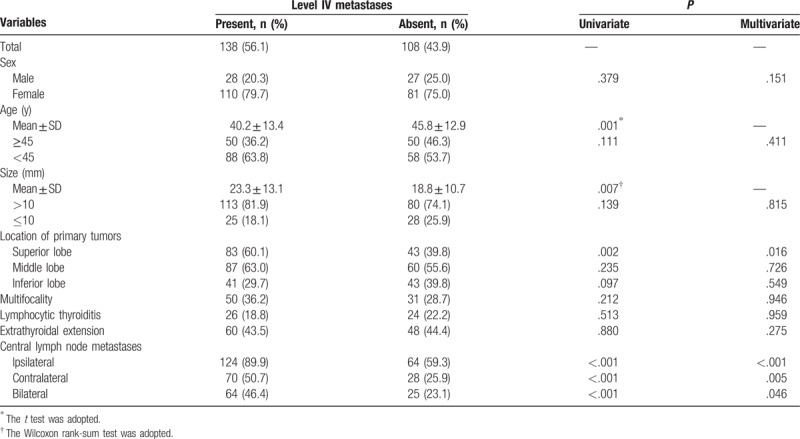
Analysis of risk factors related to level IV lymph node metastases.

**Table 6:**
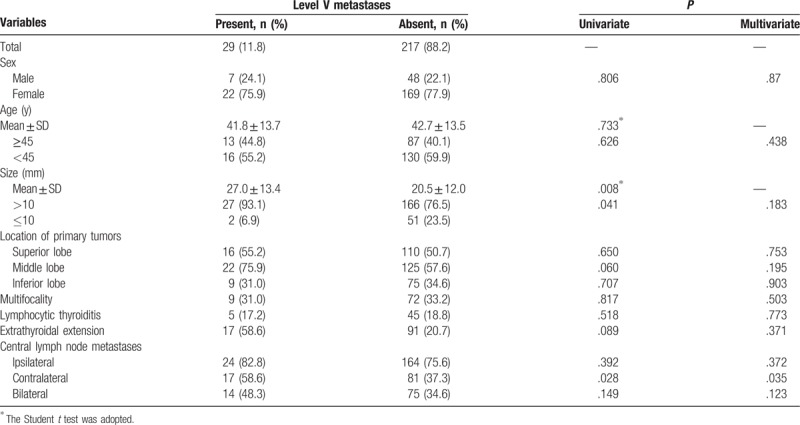
Analysis of risk factors related to level V lymph node metastases.

### 3.2 Frequency and patterns of lymph node metastases

Lymph node metastasis was histologically confirmed to involve the central compartment in 196 patients (79.7%) and the lateral compartment in 199 patients (80.9%). Of the 196 patients with CLNM, 188 patients (76.4%) had LNM in the central compartment ipsilateral to the largest primary tumor, 98 (39.8%) in the contralateral central compartment, and 89 (36.2%) in bilateral central compartments. Of the 199 patients with LLNM, 48 patients (19.5%) had single-level metastases and 151 patients (61.4%) had multilevel metastases. Level III metastases were most common (154/246; 62.6%), followed by level IV (138/246; 56.1%), level II (113/246; 45.9%), and level V (29/246; 11.8%) (Table 1).

### 3.3 Clinicopathologic risk factors for gross lateral lymph node metastases

Univariate analysis significantly showed that the presence of gross LLNM was positively associated with tumor size >10 mm, tumors located in the superior and middle lobes, extrathyroidal extension, and ipsilateral, contralateral, and bilateral CLNM, and negatively associated with tumors in the inferior lobe. Sex, age, multifocality, and coexisting lymphocytic thyroiditis were not significantly associated with the presence of LLNM. Multivariate analysis significantly showed that the LLNM was positively associated with tumors located in the superior lobe, extrathyroidal extension, and ipsilateral and bilateral CLNM.

### 3.4 Clinicopathologic risk factors for lateral lymph node metastases at each level

Univariate analysis significantly showed that the presence of level II LNM was positively associated with age ≥45 years, tumors located in the inferior lobe, extrathyroidal extension, and CLNM (ipsilateral, contralateral, and bilateral), and negatively associated with tumors located in the superior lobe (Table 3). Level III LNM showed a significant positive association with tumor size >10 mm, tumors located in the middle and inferior lobes, extrathyroidal extension, and CLNM (ipsilateral, contralateral and bilateral), and a significant negative association with tumors located in the superior lobe (Table 4). Level IV LNM showed a significant positive association with tumors located in the inferior lobe and CLNM (ipsilateral, contralateral, and bilateral) (Table 5). Level V LNM showed a significant positive association with tumor size >10 mm and contralateral CLNM (Table 6).

On multivariate analysis, age ≥45 years, tumors located in the superior lobe, extrathyroidal extension, and ipsilateral and contralateral CLNM were independent risk factors for level II LNM (Table 2). Age ≥45 years, tumors located in the superior and middle lobes, extrathyroidal extension, and ipsilateral CLNM were independent risk factors for level III LNM (Table 3). Tumors located in the superior lobe and ipsilateral, contralateral, and bilateral CLNM were independent risk factors for level IV LNM (Table 4). Only contralateral CLNM was an independent risk factor for level V LNM (Table 5).

## 4 Discussion

The effect of local LNM on survival in patients with PTC remains unclear. However, the presence of LNM significantly increases the risk of locoregional recurrence.^[4–7]^ Large population-based studies have identified a decrease in disease-free survival rate and an increase in mortality in patients with regional LNM.^[7–10]^ There is a universal agreement that therapeutic LND should be performed in patients with PTC.^[13,14]^ However, the optimal extent of therapeutic LND remains controversial. The American Thyroid Association guidelines advocate compartment-oriented en bloc LND in patients with clinical LLNM, but offer no recommendation concerning which nodal levels should be dissected.^[21]^ Therefore, this study aimed to explore the frequency and pattern of and the clinicopathologic risk factors for LLNM of each nodal level in PTC patients with clinical LLNM, to determine the rational extent of therapeutic LND.

Previous studies^[20,22–24]^ have indicated that in patients with PTC and clinical LNM, most LLNM were levels II, III, and IV, and presented at multiple level. This is consistent with the findings of the present study, which found that LLNM mainly occurred in level II, III, and IV with frequencies of 45.9%, 62.6%, and 56.1%, respectively. Based on the high prevalence of LLNM, the prognostic significance multilevel metastases, it is universally agreed that levels II to IV should routinely included in therapeutic LND. However, the extent of level II (IIa and IIb) LLNM remains unclear. Level II LNM specimens were not subdivided into levels IIa and IIb in the present study. Level V LNM were the least frequent (11.8%), which agrees reasonably well with previously reported frequencies of 6% to 29%.^[22–25]^ So, it remains controversial whether routine level V dissection should be included in therapeutic LND. The risk of postoperative complications (including injury of the spinal accessory nerve and cervical plexus, chyle leak, pain, and shoulder dysfunction) leading to increasing morbidity and worse quality of life increases with extension of radical LND. Therefore, the need to perform routine level V dissection has been questioned.^[15–18]^ Furthermore, because level V LNM are comparatively rare, some researchers have suggested that routine level V dissection is not necessary for patients with PTC and lateral cervical lymphadenopathy.^[26,27]^ However, other authors hold the opposite view based on high rates of metastasis.^[28–30]^ Based on the low frequency of level V LLNM in the present study and the risk of postoperative complications, we propose that therapeutic LND should not routinely include level V dissection, unless level V LLNM is suspicious based on preoperative examination or associated risk factors.

Most previous studies of PTC patients with lateral cervical lymphadenopathy have explored the risk factors for LLNM without separating each level or with focusing only on level II or/and level V.^[20,23–32]^ Furthermore, there are few studies that investigate CLNM with separation into ipsilateral, contralateral, and bilateral compartments to explore the risk factors of LLNM in PTC patients with lateral cervical lymphadenopathy. Koo et al^[33]^ only explored the presence of LLNM to predict occult contralateral central LNM. However, it is unclear whether the presence of contralateral CLNM is a risk factor for LLNM. The present study explored the risk factors for not only gross LLNM but also level II to IV LNM in PTC patients with lateral cervical lymphadenopathy.

The findings of the present study suggested that the mean age and tumor size of patients with gross LLNM were larger compared with those of patients without LLNM, and tumors located in the superior lobe, extrathyroidal extension, and ipsilateral and contralateral CLNM were independent risk factors for LLNM with multivariate analysis. Kwak et al^[34]^ and Zeng et al^[32]^ also found that tumors located in the superior lobe were a dependent risk factor for LLNM. However, Keum et al^[20]^ found no significant association between tumor location and LLNM. Similar to the present study, Girardi et al^[35]^ found that extrathyroidal extension was an independent risk factor for LLNM; however, the study reported by Keum et al^[20]^ did not support this finding. Many studies have shown that the presence of CLNM is an independent risk factor for LLNM.^[23,34,36]^ In the present study, both ipsilateral and contralateral CLNM were independent risk factors for LLNM, but bilateral CLNM was not. If there is no ipsilateral CLNM, the presence of contralertral CLNM is also a predictive factor for LLNM, and vice versa. However, Lim et al^[36]^ found that bilateral CLNM was an independent risk factor for occult LLNM. The mean tumor size of patients with LLNM was larger than that of patients without LLNM in the present study. Tumor size >10 mm and tumors located in the middle lobe were associated with LLNM, but were not independent risk factors for LLNM. Similar to the present study, Hunt et al^[19]^ found that patients with LLNM were younger compared with patients without LLNM. However, age ≥45 years was not an independent risk factor for LLNM. The presence of gross LLNM was not significantly associated with other clinicopathologic factors (sex, multifocality, and coexisting lymphocytic thyroiditis). Nam et al^[23]^ and Girardi et al^[35]^ found that male sex was an independent risk factor for LLNM. Zeng et al^[32]^ found that coexisting Hashimoto thyroiditis (HT) was an independent predictive factor for LLNM. Conversely, a meta-analysis by Lee et al^[37]^ suggested that PTCs with coexisting HT had a significant negative association with LNM (odds ratio [OR] 1.3, *P* = .041); however, a subgroup analysis of central of LLNM was not performed. The majority of studies found no association between multifocality and LLNM.^[19,23,34–36]^ Interestingly, the present study found that tumor located in the inferior lobe was negatively associated with LLNM, indicating that LLNM are less likely to occur in patients with PTC located in the inferior lobe.

Analysis of the risk factors for LLNM of each level revealed some diverse outcomes, especially for level V LNM. Sex was not associated with LLNM of any level. Patients with level II, III, and IV LNM were younger and had larger tumors compared with patients without LLNM. However, while patients with level V LNM still had larger tumors, they have close age compared with patients without LNM. Most of the risk factors for gross LLNM were the same as the risk factors for level II LNM. However, the risk factors differed in that age ≥45 years was an independent risk factor for level II LNM and there was no statistically significant association between tumor size >10 mm and level II LNM. For level III LNM, age ≥45 years, tumors located in the superior and middle lobes, extrathyroidal extension, and ipsilateral CLNM were independent risk factors. For level IV LNM, tumors located in the superior lobes and all CLNM (ipsilateral, contralateral, and bilateral) were independent risk factors. For level V LNM, tumor size >10 mm and contralateral CLNM were associated with LNM, but only contralateral CLNM was an independent risk factor. However, Shim et al^[27]^ and Zhang et al^[30]^ found that extrathyroidal extension was an independent risk factor for level V LNM. Therefore, the extent of CND should be carefully evaluated for different levels based on the respective independent risk factors. For example, ipsilateral lateral lymphadenopathy on preoperative examination in PTC patients with tumors located in the superior lobe would indicate that least level II to IV LND would be appropriate. In patients with no clinical evidence of level V LNM, but with contralateral CLNM, level V LND may be additionally considered.

There are some potential limitations in the present study. This was a retrospective study based on the review of pathological reports. Levels IIa and IIb, and levels Va and Vb could not be assessed. Furthermore, contralateral CLNM may be affected by multifocality, but risk factors for LLNM in patients with single or multifocal tumors were not separately investigated because it was difficult to determine whether the multifocality resulted from spread of the primary tumor or new tumor growth. Some clinicopathologic risk factors such as histological subtype, lymphovascular invasion, and distant metastasis were not included because they were not routinely reported in the pathological report. Finally, patients who underwent bilateral, elective therapeutic, or prophylactic LND were not enrolled, which may weaken the solid outcomes.

In conclusion, the present study found a relatively high prevalence of level II to IV LNM and a low prevalence of level V LNM in PTC patients with lateral cervical lymphadenopathy. Age ≥45 years, tumors located in the superior lobe, extrathyroidal extension, and ipsilateral and contralertral CLNM were independent risk factors for level II LNM. Apart from this, age ≥45 years, tumors located in the superior and middle lobes, extrathyroidal extension, and ipsilateral CLNM were independent risk factors for level III LNM. Also, tumors located in the superior lobe and ipsilateral, contralertral, and bilateral CLNM were independent risk factors for level IV LNM. For level V LNM, only contralateral CLNM was an independent risk factor. These findings are beneficial for surgeon to select the appropriate extent of LND. However, further prospective studies are extremely needed to test the present conclusion.

## Author contributions

**Conceptualization:** Zhihui Li.

**Data curation:** Shuping Yan.

**Formal analysis:** Anping Su.

**Investigation:** Anping Su.

**Methodology:** Jing Yang.

**Project administration:** Feng Liu.

**Resources:** Jing Yang.

**Software:** Jing Yang.

**Supervision:** Rixiang Gong, Jingqiang Zhu.

**Validation:** Rixiang Gong, Jingqiang Zhu.

**Visualization:** Rixiang Gong, Jingqiang Zhu.

**Writing – original draft:** Yanping Gong.

**Writing – review & editing:** Yanping Gong, Zhihui Li.
